# Monthly Summary

**Published:** 1886-08

**Authors:** 


### Monthly Summary.

---

A DISAGREEABLE ACCIDENT CONNECTED WITH THE EXTRACTION OF A TOOTH.—*By Hartman, Dentist, Barmen.* —A few days ago a man called and requested me to remove the left wisdom tooth of the lower jaw. For loosening those teeth I am in the habit of using the lever of *Lecluse*, and taking my position as far as the operating chair permits, in the front of the patient. The tooth did not yield to a slight pressure; applying a stronger one, I sustained from the man, with his right leg, such a kick upon my knee, that a quick and vehement movement on my part was caused and the tooth broke off. But the patient did not spit out the crown of the tooth, and besides I could not discover any trace of it in his mouth. The patient having cleansed his mouth from blood, by rinsing it repeatedly, asserted that he felt something sharp in his throat, which was exerting pressure, and incited coughing. The mouth being meanwhile thoroughly cleansed, I looked into it more closely, and discovered the crown of the tooth imbedded in the (velum palati) soft part of the palate, presenting its masticating surface to me. With a pair of pincers I cautiously removed the fragment, and found a small quadrangular hole in the soft palate, corresponding to the form of the tooth. The tooth was broken off by the false pressure I had involuntarily exerted with great vehemence, it had turned over, cutting out with its sharp edge, a piece of flesh. Thus it had exercised a similar effect, as when a pistol-ball is shot through a window pane.

I had to let the man withdraw, without doing anything more, as he would not submit to my adopting any further treatment. He did not keep his promise of calling again, so I am not aware of what has become of the hole in the velum. I publish my accident with the object of admonishing practitioners to exert great caution in the use of the lever.—*Deutsche Monatsschrift fur Zalmheilkunde.*

---

ALL PORCELAIN CROWNS AND BRIDGE WORK.—*By E. Parmly Brown, D. D. S., Flushing, N. Y.**—The era of removable artificial dentures is soon to pass away—excepting in rare instances, and for the very poor. The development of tooth filling to a state of great perfection is nipping caries in the bud. Badly decayed teeth are being crowned with porcelain and gold crowns, preserving them better at that advanced stage of injury than the former practice of filling, not speaking of the greater art in the practice where porcelain crowns are

*Read before the Dental Society of the State of New York, May, '86.

used, and the saving of nerve force and time to both patient and operator.

The total loss ot from one to ten teeth on a single jaw is being remedied by permanent fixtures that, I am anxious to be put on record as saying, are in five important respects better than the removable dentures of metals and plastics that are commonly used for the purpose:

Highet art can be attained—greater strength.

As perfect cleanliness as the natural teeth at their best.

A more normal condition of the tissues of the mouth than with plates that cover the mucous surfaces.

Far greater comfort to the wearer, with an ability to masticate the food only excelled by the natural teeth themselves in a good condition; the taste not being interfered with by the roof of the mouth being wholly or partly covered as with upper removable dentures; the remaining teeth not being injured one quarter as much with the permanent fixtures as with removable plates.

And last, the great satisfaction the patient expresses in not having a movable artificial plate in the mouth.

These are strong assertions some of you will say, but they are more than assertions. They have been demonstrated to my entire satisfaction by several years practice, and I am ready and anxious to demonstrate them to you, and prove each and every one of the claims I make as you may demand.

The all porcelain crowns and bridge work invented and practiced by me is the kind of work I refer to, and the only kind that will bear me out in the assertions of greater truth to nature, greater cleanliness and greater strength.*

Let the better element in the profession hail with delight any changes that lift us out of the slaughter-house, tooth-pulling era of the nitrous oxide gas introduction.

Let them hail with delight any changes that lift us above the mule-driving, hod-carrying ability necessary to construct the five dollar rubber set of gum sections, that usually looks like a white picket fence around a cemetery plot.—*The Odontographic Journal.*

---

*Specimens were exhibited and described.

THE DESTRUCTIVE ENERGY OF THE TINCTURE OF THE CHLORIDE OF IRON ON THE TEETH.—An original paper of conspicuous merit with the above title was read before the New York Odontological Society, by George W. Weld, M. D., D. D. S.

As the researches of Dr. Weld in this direction possess many points worthy of the careful consideration of every dentist and physician, the salient features of the paper are cheerfully published.

Dr. Weld declares that clinical observation shows that water increases the destructive energy of the tincture of the chloride of iron upon the enamel of the teeth more than any other fluid, and as an illustration he states that the effect of adding water to a simple solution of the chloride of iron, *devoid of free acid*, is to give basic salts of iron and the separation of free hydrochloric acid.

Dr. Weld showed conclusively that the tincture of the chloride of iron of the official strength had but little, if any, effect upon the enamel structure of a tooth, when immersed in the same for a period of twelve hours, but that when immersed in a solution of the tincture and water in proportion of one ounce of water to one drachm of the tincture, the enamel was materially injured in five minutes.

As an illustration of this phenomenon the Doctor stated that, when a piece of zinc is immersed in strong sulphuric acid ($H_2$, $SO_4$,) it has been observed that the acids has no effect upon the structure of the zinc, but if a little water be added to the acid the zinc is at once destroyed. So that it is not entirely a matter of the strength of the fluids, so far as the quantity of iron or acid is concerned but a matter of constitution or solubility.

The zinc in the strong sulphuric acid is protected in the same manner that the tooth which is immersed in the strong tincture of the chloride of iron is protected, viz: The surface is blackened up with the basic salts of iron insoluble in alcohol, and which prevents chemical action. In the case of the zinc it is the sulphate of zinc resulting from the first action, and insoluble in the concentrated acid, that forms a protecting coat over the surface of the zinc; the addition of water dissolves this protecting sulphate, and renders further chemical action possible. In the case of a tooth immersed in the strong solution of the tincture a similar action takes place, viz: the oxide of iron first formed protects the enamel from immediate chemical action on account of its compact adherence to its surface.

To illustrate still further, Dr. Weld called attention to two specimens of teeth on the card*which had been immersed in the tincture and alcohol, and compared them with teeth which had been immersed in the tincture and water. Here it was observed that although the alcoholic solution used contained the same quantity of the tincture and possessed apparently the same relative strength and immersed for the same length of time, yet no injurious effect was produced on the lime-salts of the teeth. The reason is attributed to the fact that alcohol is a dehydrating compound and that the peroxide which is formed in the alcoholic solution is of the anhydrous form, and in character very compact, adhering closely to the surface of the tooth, thereby preventing immediate chemical action, whilst on the other hand, in the presence of water the peroxide† which is precipitated is the hydrated form and flocculent in character, does not so well adhere to the surface of the tooth, leaving the free hydrochloric acid in the solution to unite with the lime-salts with greater facility.

There appears then to be two forms of the peroxide or iron, viz: (1) The hydrated form ($Fe_2(OH)6$)formed in the water solution, which is flocculent and non-protecting to the teeth. (2) The anhydrous form $Fe_2(O_3)$ formed in the alcoholic solution, which is heavy and compact, and protects the surfaces of the teeth.

The following formula will show how the hydrated peroxide is formed from the anhydrous peroxide :($Fe_2O_3 + 3H_2O = Fe_2(OH)6$).

The teeth immersed in an ounce of the elixir of the pyrophosphate of iron with one drachm of the tincture of the

*A glass case presented to the Odontological Society containing sixty-four teeth, showing, with various modifications, the destructive energy of different acids and iron compounds on the enamel.

†Synonyms: Ferric Hydroxide. Hydrated sexqu-oxide of iron.

chloride added for a period of twenty four hours, produced apparently no chemical effect on the enamel; but with the same quantity of water and the tincture the enamel was completely destroyed. The elixirs are composed of nearly twenty-five per cent. of alcohol, the presence of which, as observed in the strong solution of the tincture, and in the alcoholic solution, affords a protection to the enamel of the teeth in the manner described. But it is to be noted that when a tooth is immeresd in a solution of the tincture and simple syrup, in the above proportions, the enamel is but little affected This is due to a mechanical reason or a condition of fluidity of the solution, i. e., the presence of the sugar in solution coats the surface of the enamel, preventing the chemical affinity between the acid, or perchloride of iron, and the lime-salts in the teeth.

The manner in which syrup modifies the destructive energy of the tincture on the enamel was beautifully illustrated by the effect produced on the specimens of teeth which had been immersed in a weak solution of phosphoric acid, viz. "Hosford's acid Phosphate," "Phos-acid," and "Phospho-Muriate of Quinine Compound." The first two of these proprietary medicines are water solutions, and the effect is to destroy the enamel of a tooth in an hour, whilst the last one, which is a syrup solution (each fluid drachm containing two grains of free phosphoric acid) produces but little, if any, injurious effect on the enamel in twenty-four hours.

Equally interesting was the effect produced on the enamel of the teeth which had been immersed in a solution of the tincture and the weak alkaline waters (notably Vichy.)

When a drachm of the tincture is added to an ounce of Vichy-water, a slight effervescence occurs, indicating that the bicarbonate of soda contained in the water has neutralized a part of the free acid contained in the tincture; iu consequence, when a tooth is immersed in such a solution, the destructive energy of the iron is to a great extent modified. Unless the specific nature of the tincture of the chloride of iron is materially affected (and the peculiar odor of the tincture remains) there seems to be no reason why this preparation of iron, at least in all cases of anæmia, should not be administered in combination with Vichy-water.

There are then three menstrua which may be employed to modify the destructive energy of the tincture of the chloride of iron on the enamel of the human teeth. The first is alcohol in some form. The second is Vichy-water, which neutralizes to a slight extent the free acid contained in the iron. And the third is some form of an elixir or simple syrup.—*Odontographic Journal.*

